# The determinants of complication trajectories in American Indians with type 2 diabetes

**DOI:** 10.1172/jci.insight.146849

**Published:** 2021-05-24

**Authors:** Evan L. Reynolds, Gulcin Akinci, Mousumi Banerjee, Helen C. Looker, Adam Patterson, Robert G. Nelson, Eva L. Feldman, Brian C. Callaghan

**Affiliations:** 1 Department of Neurology, University of Michigan, Ann Arbor, Michigan, USA.; 2 Division of Pediatric Neurology, Dr. Behcet Uz Children’s Hospital, Izmir, Turkey.; 3 Department of Biostatistics, University of Michigan, Ann Arbor, Michigan, USA.; 4 Chronic Kidney Disease Section, National Institute of Diabetes and Digestive and Kidney Diseases (NIDDK), Phoenix, Arizona, USA.

**Keywords:** Endocrinology, Chronic kidney disease, Diabetes, Neuromuscular disease

## Abstract

**BACKGROUND:**

We aimed to determine whether metabolic syndrome (MetS) affects longitudinal trajectories of diabetic complications, including neuropathy, cardiovascular autonomic neuropathy (CAN), and kidney disease in American Indians with type 2 diabetes.

**METHODS:**

We performed a prospective study where participants underwent annual metabolic phenotyping and outcome measurements. The updated National Cholesterol Education Program criteria were used to define MetS and its individual components, using BMI instead of waist circumference. Neuropathy was defined using the Michigan Neuropathy Screening Instrument index, CAN with the expiration/inspiration ratio, and kidney disease with glomerular filtration rate. Mixed-effects models were used to evaluate associations between MetS and these outcomes.

**RESULTS:**

We enrolled 141 participants: 73.1% female, a mean (±SD) age of 49.8 (12.3), and a diabetes duration of 19.6 years (9.7 years) who were followed for a mean of 3.1 years (1.7 years). MetS components were stable during follow-up except for declining obesity and cholesterol. Neuropathy (point estimate [PE]: 0.30, 95% CI: 0.24, 0.35) and kidney disease (PE: –14.2, 95% CI: –16.8, –11.4) worsened over time, but CAN did not (PE: –0.002, 95% CI: –0.006, 0.002). We found a significant interaction between the number of MetS components and time for neuropathy (PE: 0.05, 95% CI: 0.01–0.10) but not CAN (PE: –0.003, 95% CI: –0.007, 0.001) or kidney disease (PE: –0.69, 95% CI: –3.16, 1.76). Systolic blood pressure (SBP, unit = 10 mmHg) was associated with each complication: neuropathy (PE: 0.23, 95% CI: 0.07, 0.39), CAN (PE: –0.02, 95% CI: –0.03, –0.02), and kidney disease (PE: –10.2, 95% CI: –15.4, –5.1).

**CONCLUSION:**

In participants with longstanding diabetes, neuropathy and kidney disease worsened during follow-up, despite stable to improving MetS components, suggesting that early metabolic intervention is necessary to prevent complications in such patients. Additionally, the number of MetS components was associated with an increased rate of neuropathy progression, and SBP was associated with each complication.

**FUNDING:**

The following are funding sources: NIH T32NS0007222, NIH R24DK082841, NIH R21NS102924, NIH R01DK115687, the Intramural Program of the NIDDK, the NeuroNetwork for Emerging Therapies, the Robert and Katherine Jacobs Environmental Health Initiative, the Robert E. Nederlander Sr. Program for Alzheimer’s Research, and the Sinai Medical Staff Foundation.

**TRIAL REGISTRATION:**

ClinicalTrials.gov, NCT00340678.

## Introduction

Pima Indians have a disproportionately high prevalence of type 2 diabetes with an earlier onset compared with the general population (1–5). In addition to hyperglycemia, other components of the metabolic syndrome (MetS), such as obesity, low HDL cholesterol, and elevated triglycerides are common in this population (4, 6–9). Furthermore, Pima Indians have elevated rates of diabetic complications, including incidence of kidney disease and prevalence of neuropathy (10, 11). Therefore, Pima Indians are the ideal population to investigate the associations between MetS and its individual components on diabetic complications.

Multiple cross-sectional, population-based studies have revealed an association between MetS and neuropathy (12–19). After diabetes, obesity has emerged as the second most important individual MetS component associated with neuropathy (16–18, 20–26). Furthermore, multiple studies have found an increasing prevalence of neuropathy as the number of MetS components increases (14, 16, 18, 20, 27). The association between MetS and cardiovascular autonomic neuropathy (CAN) has been less extensively studied; however, multiple investigators have revealed associations between individual MetS components and CAN, with obesity and high blood pressure as common risk factors (28–36). Similarly, a meta-analysis outlined several studies that found significant associations between MetS and kidney disease and that each MetS component was associated with a higher risk of kidney disease (37–50). Some of these studies found increasing risk of kidney disease as the number of MetS components increased (41, 42, 44, 49). Recently, we assessed the effects of the MetS on diabetic complications, including neuropathy, CAN, and kidney disease (20, 31). We found that for neuropathy and CAN, after diabetes, central obesity was the most important individual risk factor for disease. In contrast, obesity alone was not associated with kidney disease.

Previous studies have primarily been cross-sectional; therefore, it is unclear how MetS components and diabetic complications change over time. It is also unknown whether MetS affects the rate by which diabetic complications worsen over time. Additionally, more evidence is needed to determine the association between neuropathy, CAN, kidney disease, and individual MetS components, especially in a population with a high prevalence of diabetic complications and MetS. In this study, we aimed to determine the longitudinal change of MetS components and diabetic complication severity in a cohort of Pima Indians with type 2 diabetes. We also investigated whether the number of MetS components was associated with differential rates of diabetic complication severity. Lastly, we investigated whether any individual MetS components were associated with neuropathy, CAN, and/or kidney disease.

## Results

### Population and description of follow-up.

Between 2013 and 2019, 141 Pima Indians were followed for a mean (SD) duration of 3.1 (1.7) years. Most incomplete follow-up was due to differential study enrollment date. Specifically, there were 101 (71.6%) participants who completed longitudinal follow-up during the study period, and 40 participants who were lost to follow-up.

### Demographic information and longitudinal change in metabolic risk factors.

Demographic and metabolic phenotyping at baseline and in each year of follow-up are presented in Table 1. At study entry, 73.1% of participants were female and 26.9% were male. Participants had a mean (SD) age of 49.8 (12.3) years and had diabetes for an average of 19.6 (9.7) years. During follow-up, participant systolic blood pressure (SBP) at baseline was 119.8 (14.7) and at 3 years 122.6 (16.2), *n* = 88; diastolic blood pressure (DBP) at baseline was 71.9 (8.9) and at 3 years 73.4 (10.5), *n* = 88; HbA1c at baseline was 9.6 (2.2) and at 3 years 9.1 (2.1), *n* = 83; triglycerides at baseline were 147.2 (92.7) and at 3 years 168.6 (95.1), *n* = 84; and total cholesterol at baseline was 149.0 (41.7) and at 3 years 151.5 (41.4), *n* = 84; none of these measures significantly changed (all *P* > 0.05). However, weight decreased from 95.7 (23.7) at baseline to 91.8 (21.3) at 3 years, *n* = 88; HDL increased from 41.6 (11.9) at baseline to 44.7 (9.9) at 3 years, *n* = 83; and LDL decreased from 86.8 (33.1) at baseline to 74.1 (32.1) at 3 years, *n* = 82 (all *P* < 0.05). At baseline, no participants were receiving fibrates or niacin for high cholesterol, and 62.4% of participants were receiving medication for hypertension.

### Longitudinal change in primary measures of neuropathy, CAN, and kidney disease.

**Figure 1:**
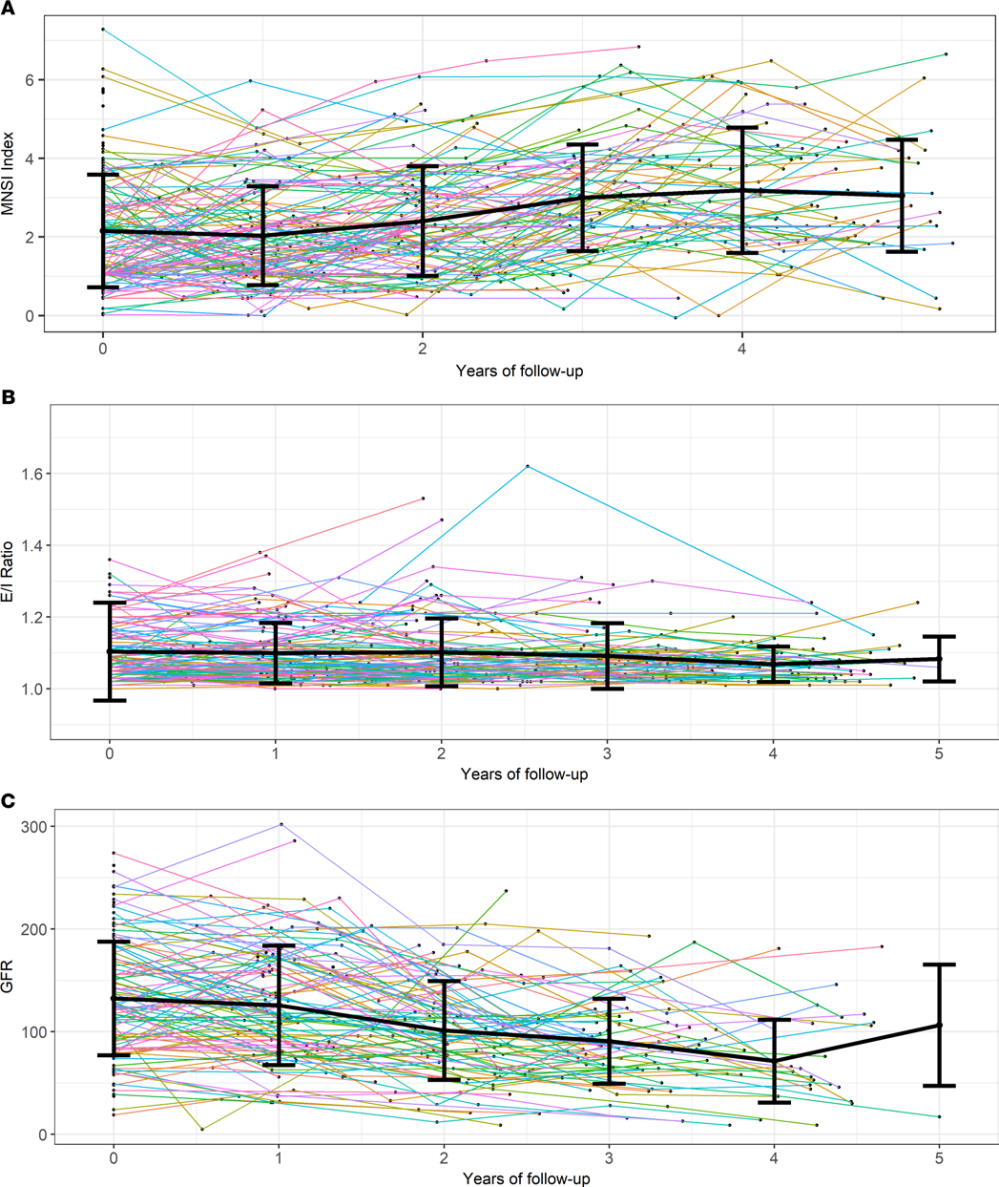
Participant outcomes during follow-up. (**A**) Participant-specific neuropathy (MNSI index). (**B**) CAN (E/I ratio). (**C**) Kidney disease (GFR). Data shown as mean ± SD. Error bars represent the SD for the outcome measurements within the nearest year of follow-up. The number of participants with outcome measurements in each nearest year of follow-up for MNSI index at baseline: 140, year 1: 117, year 2: 106, year 3: 87, year 4: 59, year 5: 38; for E/I ratio, baseline: 136, year 1: 115, year 2: 96, year 3: 75, year 4: 56, year 5: 13; and for GFR, baseline: 126, year 1: 107, year 2: 86, year 3: 63, year 4: 30, year 5: 5. One outlier was removed in **B** (E/I ratio = 2.0 at baseline) in order to describe participant trajectories more clearly.

**Figure 2:**
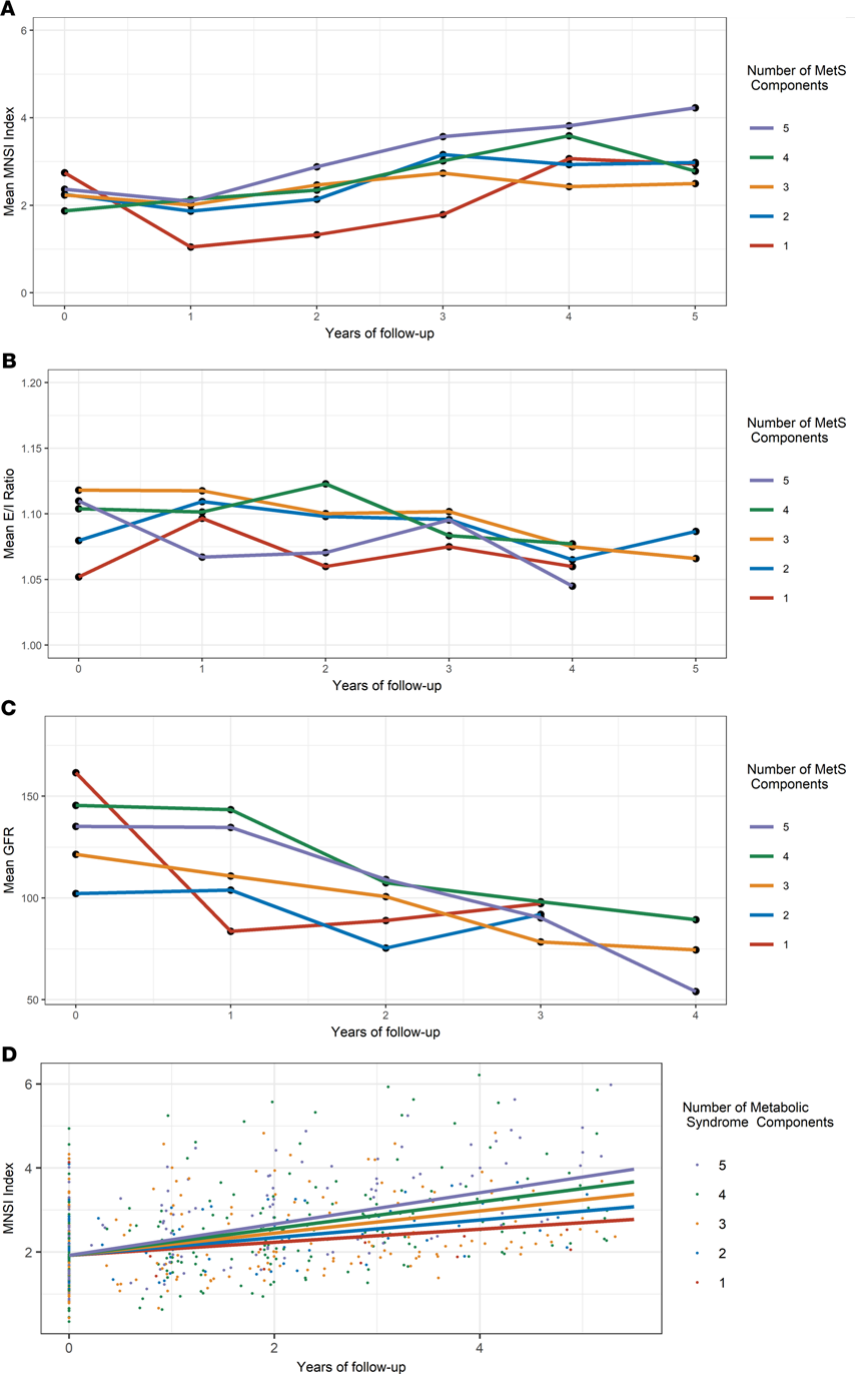
Longitudinal mean changes in neuropathy (MNSI index), CAN (E/I ratio), and kidney disease (GFR) outcomes during follow-up, stratified by the number of MetS components. (**A**) Mean neuropathy, (**B**) mean CAN, (**C**) mean GFR, and (**D**) predicted neuropathy from a linear mixed-effects model in each year of follow-up, stratified by the number of MetS components. Mean outcomes based on MetS subgroups with less than 3 participants were excluded from the figures.

Each participant’s trajectories of neuropathy, CAN, and kidney disease outcomes are displayed in Figure 1, A–C. Primary neuropathy was measured by the Michigan Neuropathy Screening Instrument (MNSI) combined index: the baseline was 2.1 (1.4) and at 3 years it was 3.0 (1.4), *P* < 0.01, *n* = 87. Kidney disease was measured by glomerular filtration rate (GFR, unit = mL/min): the baseline was 135.6 (55.7) and at 3 years it was 90.7 (41.4), *P* < 0.01, *n* = 58. Primary neuropathy and kidney disease outcomes worsened over time, but CAN did not, as measured by expiration to inspiration (E/I) ratio: at baseline 1.11 (0.16) and at 3 years 1.09 (0.09), *P* = 0.47, *n* = 70. At baseline, 20.7% of participants met the cutoff for neuropathy (MNSI index > 3.2516), which generally increased during follow-up (19.7% in year 1, 28.3% in year 2, 36.8% in year 3, 44.1% in year 4, and 39.5% in year 5). The trajectories of neuropathy, CAN, and kidney disease outcomes, stratified by the number of MetS components participants had, are displayed in Figure 2, A–C. The MNSI index increased more rapidly for participants with 5 MetS components from 2.4 (1.2) at baseline to 3.6 (1.8) at 3 years compared with patients with only diabetes with 2.8 (1.9) at baseline and 1.8 (1.2) at 3 years. In contrast, GFR decreased at similar rates across all participants, regardless of whether they had 5 MetS components, with a baseline of 135.2 (56.2) and at 3 years 90.3 (36.0); 3 MetS components, with a baseline of 121.4 (47.6) and at 3 years 78.4 (37.1); or only diabetes with a baseline of 161.6 (71.2) and at 3 years 97.3 (69.1).

### Longitudinal change in secondary measures of neuropathy, CAN, and kidney disease.

Individually, the MNSI examination significantly worsened during follow-up, with a baseline of 4.0 (1.7) and at 3 years 4.7 (1.6), *P* < 0.01, *n* = 87. The MNSI questionnaire also significantly worsened during follow-up, with a baseline of 2.8 (2.4) and at 3 years 4.1 (2.7), *P* < 0.01, *n* = 87. In contrast, there were fewer participants with an abnormal monofilament test after 3 years of follow-up, with a baseline of 44 (50.6%) and at 3 years 27 (31.0%), *P* < 0.01, *n* = 87. Secondary outcomes did not change during follow-up for CAN (autonomic symptoms profile), with a baseline of 17.9 (9.8) and at 3 years 17.3 (10.3) (*P* = 0.63, *n* = 81) or for kidney disease as measured by albumin to creatinine ratio (ACR, unit = mg/g), log(ACR), with a baseline of 3.96 (1.68) and at 3 years 4.13 (1.94) (*P* = 0.21, *n* = 83).

### Longitudinal change in quality of life.

Although neuropathy worsened during follow-up, Neuropathy-Specific Quality of Life (Neuro-QOL) measures remained largely unchanged. The mean (SD) total Neuro-QOL score did not change during follow-up from baseline of 2.2 (2.0) to 2.1 (1.8) at 3 years (*P* = 0.92, *n* = 67). Considering each Neuro-QOL subcomponent individually, only QOL regarding diffuse sensory motor symptoms with a baseline of 2.5 (2.3) and 3.1 (3.3) at 3 years (*P* < 0.01, *n* = 82) significantly worsened during follow-up, and only QOL regarding activities of daily living improved during follow-up from a baseline of 3.0 (4.2) to 1.9 (1.8) at 3 years (*P* = 0.045, *n* = 86).

### Linear mixed-effects models with individual MetS components.

Linear mixed-effects models revealed that MNSI index time point estimate (PE) worsened during follow-up (0.30, 95% CI: 0.03, 0.38), as did the GFR time PE (–14.2, 95% CI: –16.8, –11.4) but the E/I ratio did not (time PE: –0.002, 95% CI: –0.006, 0.003) (Table 2). SBP was the only MetS component associated with each outcome. Specifically, higher SBP (unit = 10 mmHg) was associated with worse MNSI index (PE: 0.23, 95% CI: 0.07, 0.39), E/I ratio (PE: –0.02, 95% CI: –0.03, –0.02), and GFR (PE: –10.1, 95% CI: –15.3, –5.0). In addition to time and SBP, weight (PE: 3.2, 95% CI: 1.7, 4.7) and HbA1c (PE: 7.0, 95% CI: 3.3, 10.6) were associated with GFR. Duration of diabetes (PE: –0.002, 95% CI: –0.003, –0.0002) and HbA1c (PE: –0.011, 95% CI: –0.018, –0.004) were also associated with the E/I ratio. Results from the sensitivity analyses, including the SD of individual MetS components during follow-up, yielded similar conclusions. In addition, the sensitivity analysis, including adjustment of the mixed-effects models for the use of antihypertensive medications at baseline, did not result in any substantive changes to our model inference.

### Linear mixed-effects models with the number of MetS components, time, and their interaction.

The linear mixed-effects models, including the number of MetS components, time, and their interaction revealed the number of MetS components at baseline was not associated with neuropathy (PE: 0.07, 95% CI: –0.12, 0.27), CAN (PE: –0.006, 95% CI: –0.019, 0.008), or kidney disease (PE: 5.60, 95% CI: –1.46, 12.70). The only condition with a significant time by number of MetS components interaction was neuropathy, signifying that an increased number of MetS components was associated with an increased rate of neuropathy progression over time (PE: 0.05, 95% CI: 0.01, 0.10). The predicted MNSI index over time based on the fixed effects, stratified by the number of MetS components, is displayed in Figure 2D.

## Discussion

In a prospective cohort study of Pima Indian participants with type 2 diabetes, we found that MetS components remained stable or improved during 5 years of follow-up. Despite this stability, neuropathy and kidney disease outcomes worsened over time, but CAN did not. These findings indicate that stability of metabolic risk factors is not enough to prevent neuropathy and kidney disease. SBP was the only individual MetS component associated with all 3 complications. The only other metabolic risk factors associated with worse outcomes were hyperglycemia measures (diabetes duration and HbA1c) with CAN. Furthermore, a higher number of MetS components was associated with an increased rate of neuropathy severity, but not with other outcomes. Therefore, the number of MetS components that an individual had determined their future neuropathy trajectory, and MetS may be a more important risk factor for neuropathy than for CAN or kidney disease.

Although participants’ metabolic profiles remained stable or improved during follow-up, neuropathy and kidney disease outcomes worsened considerably. Our results suggest that the cumulative effect of metabolic risk factors over many years, rather than contemporary fluctuations in metabolic risk factors, likely lead to nerve and kidney injury in this population. In contrast to our primary neuropathy outcome, the number of participants with abnormal monofilament testing decreased during follow-up, which warrants further investigation. Although previous studies have demonstrated worsening diabetic complications over time (51, 52), only one study has assessed the relationship between kidney disease progression and changes in metabolic risk factors over several years (39). The prospective cohort study of 10,685 Korean men found that worsening MetS over time was associated with an increased hazard of incident kidney disease during follow-up. We are unaware of any prior studies evaluating this longitudinal association for neuropathy or CAN. Given the higher prevalence of MetS as people age (53), we were surprised to see that the metabolic risk factors were stable during follow-up, with the exception of improving weight and cholesterol. One possible explanation is that the participants in our study agreed to be involved in clinical research, which may have led to a nonrepresentative population, more frequent medical care, and benefits from clinical trial participation. Alternatively, it is possible that declining weight and cholesterol levels may reflect worsening diabetes or kidney disease in this population. Previous studies in the Pima population have consistently found weight loss after the diagnosis of diabetes (6, 54). This is in contrast to several longitudinal studies outside of the Pima population, which have found stable or increased weight for patients after diabetes onset (55–57). Unfortunately, we did not collect the information necessary to determine whether these improvements in weight and cholesterol were due to intentional lifestyle changes or worsening disease. Our findings highlight that improving a poor metabolic standing late in the course of type 2 diabetes is unlikely to slow the onset of diabetic complications. To slow or reverse the rate of complications for patients with type 2 diabetes, early interventions that improve metabolic risk factors, and likely multiple metabolic risk factors, are needed.

Neuropathy and kidney disease measurements worsened during follow-up; however, the drivers of these complications were different. Neuropathy was driven by the number of MetS components, whereas kidney disease was driven by time itself (independent of the MetS components). Although previous studies have found a consistent association between the number of MetS components with neuropathy (14, 17, 18, 20, 27) and kidney disease (41, 42, 44, 49), our current study found that at baseline, the number of MetS components was not associated with neuropathy, CAN, or kidney disease. However, during follow-up, we found that the number of MetS components was associated with an increased rate of neuropathy severity, but not CAN or kidney disease. This is our second study that has found the number of MetS components was more important for neuropathy than autonomic nerve or kidney injury (31). This growing evidence suggests that improving individual MetS components for patients with diabetes could slow the rate of neuropathy onset, but this is less likely for kidney disease. Unfortunately, these results also suggest that interventions aimed at improving MetS components other than hyperglycemia are unlikely to simultaneously reverse or slow both diabetic complications.

Although MetS was associated with only the progression of neuropathy, we found that SBP was associated with each complication. For kidney disease, this result is in concert with previous studies that have consistently found an association with SBP (37, 38, 40, 42–49). For neuropathy and CAN, the association with SBP in the literature is less clear: although some studies have found an association between SBP, neuropathy (20, 21), and CAN (29, 32, 33, 36), others have failed to find such a relationship (13, 16–19, 22, 23, 28, 34, 35). Inconsistent associations between SBP, neuropathy, and CAN may be due to the high variability in blood pressure measurements (58). Since our study calculated mean SBP across a series of measurements during follow-up, we were able to more precisely determine participant SBP, which may have resulted in a clearer association. Another possible explanation for inconsistent findings between SBP and complications is that treatment of this condition differs between populations. In this study, 62.4% of patients were receiving medications for high blood pressure at baseline. A limitation of previous studies is that very few have simultaneously assessed the association between MetS and multiple diabetic complications, thereby limiting our understanding of potential risk factors for multiple complications. For populations with long-term diabetes, identifying modifiable risk factors, such as SBP, that may slow the rate of multiple complications could potentially reduce morbidity and improve QOL.

Despite growing evidence detailing an association between obesity, neuropathy (16–18, 20–26), CAN (28, 29, 31–34), and kidney disease (37), we surprisingly did not find weight to be negatively associated with any diabetic complication. One explanation was that we only measured general obesity using overall weight. Importantly, our recent studies assessed the association between 9 anthropometric measurements, neuropathy, CAN, and kidney disease in an obese population in the United States. We found that measurements of central obesity (waist circumference measured at the top of the iliac crest) had stronger associations with neuropathy and CAN compared with general obesity (overall weight) (20, 31). In that study, we also did not find an association between central obesity and kidney disease. Although we adjusted for sex in our analysis, our cohort was 73.1% female. Given the clear differences in body composition in this population (59), this imbalance may also have made it difficult to detect an association between obesity and each complication. Determining whether obesity is a risk factor for complications in patients with long-term diabetes requires future studies that assess obesity in a sex-balanced cohort using anthropometric measurements, including specific aspects of central obesity. Another possible explanation is that participants experienced weight loss during follow-up. This weight loss may represent an intentional improvement or be a result of wasting due to diabetes (6, 54) or chronic kidney disease (60). Randomized controlled trials are needed to determine whether weight loss resulting from worsening disease or intentional lifestyle changes, such as improved diet and/or exercise, is associated with differential progression of complications.

Our study has some limitations, including a small sample size, incomplete follow-up measurements, and lack of generalizability to other populations and ethnicities. Specifically, this study included participants with long-term diabetes; therefore, it is unclear whether our results are generalizable to patients with newly diagnosed diabetes. Additionally, participants had a long duration of diabetes at study entry and were followed for at most 5 years; therefore, our study was unable to measure long-term effects of MetS on progression of diabetic complications. Furthermore, this cohort exhibited stable to improved metabolic risk factors during follow-up, which may not be typical of other populations with diabetes. Differential follow-up length by disease severity is another potential limitation. On the other hand, few studies have evaluated longitudinal metabolic risk factors and diabetic complications. We also did not collect detailed anthropometric measurements, including aspects of central obesity. Our primary neuropathy measure, the MNSI index, has good but not perfect diagnostic characteristics for neuropathy (61). Furthermore, no nerve conduction studies were performed on this cohort. Lastly, E/I ratio is only one component in the battery of cardiovascular reflex tests that make up the gold standard for diagnosing CAN.

In summary, in a longitudinal study of participants having long diabetes duration, neuropathy and kidney disease worsened over time but CAN did not. The rate of progression was determined by the number of MetS components for neuropathy but not for kidney disease. This suggests that reversing MetS may stabilize the rate of nerve injury but may not slow the rate of kidney disease. Therefore, early interventions that reverse metabolic risk factors are needed to prevent the onset of complications for patients with diabetes. In addition to glycemic control, blood pressure management may play a key role in simultaneously preventing neuropathy, CAN, and kidney disease for patients with diabetes, but future intervention studies are needed.

## Methods

### Population and recruitment.

Between November 1, 2013, and July 1, 2019, 141 Pima Indians with type 2 diabetes from the Gila River Indian Community were enrolled in the study. At baseline and in each year of follow-up, participants underwent metabolic and diabetic complication phenotyping. Inclusion criteria were age 18 years or older, prior enrollment in the clinical trial “Renoprotection in Early Diabetic Nephropathy in Pima Indians” (ClinicalTrials.gov, NCT00340678) (62) or being a first degree relative of a clinical trial participant who had type 2 diabetes, and being able/willing to provide written informed consent for the study. Exclusion criteria included pregnancy, end-stage kidney disease, or illness with sufficient severity to preclude safe enrollment in the study. Follow-up length for each participant was defined as the duration of time between baseline and final MetS or outcome measurements. Participants had varying lengths of follow-up because of the timing of study enrollment and loss to follow-up.

### MetS components.

At baseline and in each subsequent year of follow-up, participants were examined after an overnight fast. Blood pressure was measured while the participant was resting in the seated position. Height and weight were measured in light clothing, and numerous laboratory tests were performed that included a lipid panel and HbA1c measurement. The onset of diabetes was determined based on serial glucose tolerance testing or from review of clinical records (63). The updated National Cholesterol Education Program criteria were used to define MetS and its individual components, with the exception of BMI, which was used in place of waist circumference because these data were not available (64). Specifically, using the mean value across longitudinal visits, the MetS criteria were a BMI of 30 kg/m^2^ or more, SBP of 130 mmHg or higher or DBP of 85 mmHg or higher, triglycerides of 150 mg/dL or greater, and HDL of 40 mg/dL or less in men or 50 mg/dL or less in women. Participants receiving medication for hypertension were considered to meet the individual MetS cutoff for high blood pressure. Participants receiving fibrates or niacin for cholesterol were considered to meet the MetS cutoff for both low HDL and high triglyceride levels.

### Peripheral neuropathy.

The primary neuropathy outcome was the MNSI index (65, 66). The MNSI index is calculated by summing the 15 individual items of the MNSI questionnaire and 4 individual items of the MNSI examination that are weighted based on each item’s relative importance for predicting definite neuropathy (66). To provide additional clinical context to our primary neuropathy measurement, we also determined the number of participants meeting the predefined cutoff for neuropathy based on the MNSI index (MNSI index > 3.2516) (66). Three secondary outcomes included the MNSI examination, MNSI questionnaire, and monofilament testing. Monofilament testing was performed with a Semmes Weinstein 5.07/10 g monofilament on the dorsum of the dominant great toe (67).

### CAN.

The primary CAN measure was the E/I ratio, 1 of 5 cardiovascular reflex tests proposed by Ewing (68), which are considered the gold standard for autonomic testing (69–71). The secondary outcome was a reduced version of the Composite Autonomic Symptom Profile Score, a concise instrument to assess autonomic symptom severity (72).

### Kidney disease.

The primary kidney disease outcome was the GFR (unit = mL/min). The secondary kidney disease outcome was the urine ACR (unit = mg/g). GFR was measured by the urinary clearance of iothalamate (73).

### QOL.

The validated Neuro-QOL instrument was used to measure neuropathy-specific QOL, with higher numbers reflecting a poorer QOL (74). The Neuro-QOL instrument measures total QOL and subcomponents specific to pain; reduced sensation; diffuse sensory motor symptoms; activities of daily living; and emotional, social, and foot-specific QOL.

### Statistics.

Descriptive statistics were used to characterize the population in terms of demographics, metabolic phenotyping, QOL, and outcomes at baseline and in each subsequent year of follow-up. Two-tailed, paired *t* tests were used to compare within-subject differences in metabolic factors and outcomes at baseline and at 3 years of follow-up because a majority of participants had 3 years of follow-up measurements. McNemar’s test was used to compare within-subject differences in abnormal monofilament tests from baseline to 3-year follow-up assessments. We also calculated descriptive statistics of outcomes (neuropathy, CAN, and kidney disease), stratified by the number of MetS components at baseline and each subsequent year of follow-up. Because follow-up visits were not exactly 1 year apart, we rounded each visit to the nearest year of follow-up to calculate descriptive statistics. If a participant had more than 1 visit in a given year of follow-up, the values were first averaged within-participant before the descriptive statistics of the cohort were assessed.

Linear mixed-effects models with random participant-specific intercepts were used to evaluate the associations between the primary outcome for neuropathy (MNSI index), CAN (E/I ratio), and kidney disease (GFR) with the individual mean MetS components. The mixed-effects models with random participant-specific intercepts allowed us to determine the association between MetS and all longitudinal outcome measurements during follow-up, while accounting for the correlation of repeated measurements within participants. We calculated the mean individual MetS components during follow-up for use in the linear models because of their stability during follow-up. Each outcome was modeled as a function of time (in years) in the study and mean longitudinal MetS components (weight, HbA1c, HDL, triglycerides, SBP) after adjusting for age, sex, height, and duration of diabetes. Given that height is an independent risk factor for neuropathy, both height and weight (rather than BMI), were included as individual parameters in the mixed-effects models (75). This allowed us to directly adjust for the independent effects of height when assessing the association between MetS and each outcome. As a sensitivity analysis, we also included the SD of the longitudinal MetS components (in addition to the mean values) as covariates in the mixed-effects models. In an additional sensitivity analysis, the use of antihypertensive medications at baseline (yes/no) was added to the mixed-effects models to determine the adjusted association between SBP and each outcome.

To determine whether the number of MetS (#MetS) components was associated with differential rates of outcome severity, we fit separate linear mixed-effects models, with a random participant-specific intercept, time (in years) on the study, #MetS, and a time by #MetS interaction term after adjusting for age, sex, height, and duration of diabetes.

For all hypothesis testing, available-case analysis was performed, 2-tailed *P* values were calculated, and statistical significance was determined using a *P* value threshold of 0.05. All analyses were performed using R version 3.4.2.

### Study approval.

This study was approved by the IRBs of the NIDDK and the University of Michigan. Additionally, informed written consent was obtained from each study participant.

## Author contributions

ELR was involved in the study design, performance, and interpretation of the statistical analysis and wrote the manuscript. GA was integrally involved in the interpretation of the data and critical revisions of the manuscript. MB was integrally involved in the study design, interpretation of the data, and critical revisions of the manuscript. HCL was involved in the data management and critical revisions of the manuscript. AP was involved in the data management and critical revisions of the manuscript. RGN was integrally involved in the study design, interpretation of the data, and critical revisions of the manuscript. ELF was integrally involved in obtaining the study funding, training the research coordinators, designing the study, interpreting the data, and critically revising the manuscript. BCC was involved in the study design, interpretation of the statistical analysis, and critical revisions of the manuscript.

## Supplementary Material

Trial reporting checklists

ICMJE disclosure forms

## Figures and Tables

**Table 1:**
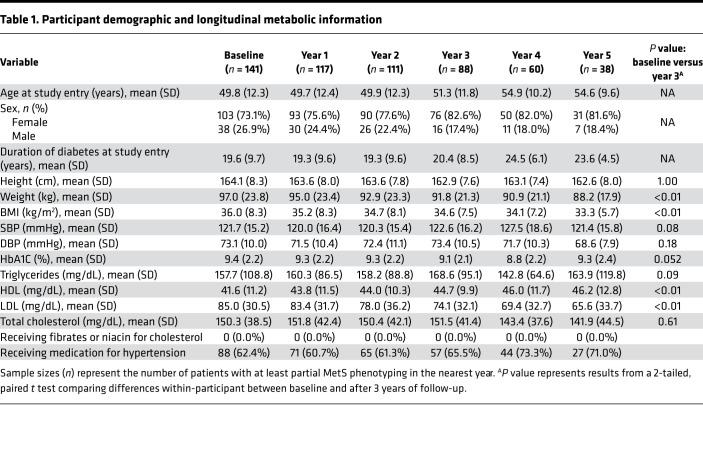
Participant demographic and longitudinal metabolic information

**Table 2:**
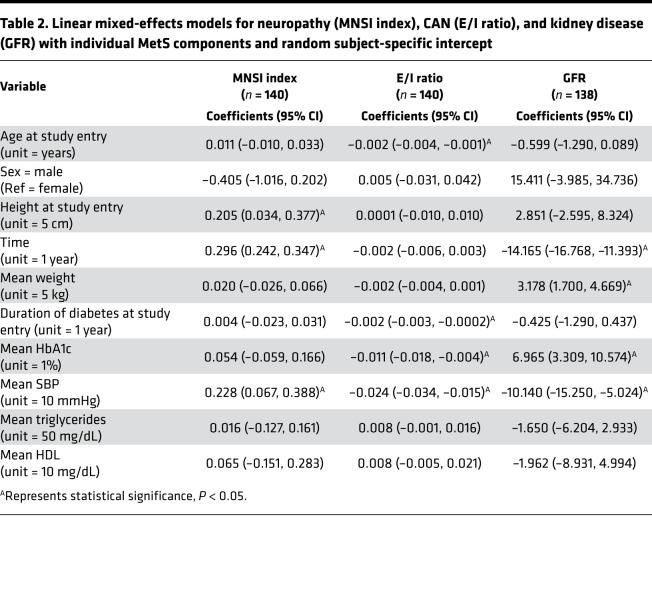
Linear mixed-effects models for neuropathy (MNSI index), CAN (E/I ratio), and kidney disease (GFR) with individual MetS components and random subject-specific intercept

## Version 1 05/24/2021

Electronic publication
