# The determinants of complication trajectories in American Indians with type 2 diabetes

**DOI:** 10.1172/jci.insight.168732

**Published:** 2023-02-08

**Authors:** Evan L. Reynolds, Gulcin Akinci, Mousumi Banerjee, Helen C. Looker, Adam Patterson, Robert G. Nelson, Eva L. Feldman, Brian C. Callaghan

Original citation: *JCI Insight*. 2021;6(10):146849. https://doi.org/10.1172/jci.insight.146849

Citation for this corrigendum: *JCI Insight*. 2023;8(3):168732. https://doi.org/10.1172/jci.insight.168732

For this article, we used the Michigan Neuropathy Screening Instrument combined index (MNSI index) to measure neuropathy longitudinally in 141 Pima American Indians. We were contacted by the authors who developed the MNSI index and were notified of an error in their published manuscript (1, 2).

Specifically, the cutoff reported by Herman et al. to determine neuropathy was incorrect. The corrected cutoff for neuropathy is MNSI index > 2.5407, rather than MNSI index > 3.2516. While we primarily used the continuous MNSI index in our paper, we also reported the prevalence that met this cutoff during each year of follow-up. In light of this information, we reanalyzed our data with the corrected cutoff.

The text in Results and Methods is corrected as follows:

At baseline, 32.9% of participants met the cutoff for neuropathy (MNSI index > 2.5407 ), which generally increased during follow-up (27.4% in year 1, 40.6% in year 2, 59.8% in year 3, 57.6% in year 4, and 55.3% in year 5).

To provide additional clinical context to our primary neuropathy measurement, we also determined the number of participants meeting the predefined cutoff for neuropathy based on the MNSI index (MNSI index > 2.5407) (66).

## Version 1 02/08/2023

Electronic publication
