# Supplementary material for: The determinants of complication trajectories in American Indians with type 2 diabetes
Source: JCI Insight. 2021 May 24;6(10):e146849. doi: 10.1172/jci.insight.146849 (PMC8262294; doi:10.1172/jci.insight.146849)
Supplement: Trial reporting checklists [file jciinsight-6-146849-s112.pdf]

STROBE Statement—Checklist of items that should be included in reports of ***cohort studies***

	Item No	Recommendation
**Title and abstract**	1	(*a*) Indicate the study's design with a commonly used term in the title or the abstract (Page 3, lines 98-99)
		(*b*) Provide in the abstract an informative and balanced summary of what was done and what was found (Page 3, lines 106-115)
**Introduction**		
Background/rationale	2	Explain the scientific background and rationale for the investigation being reported (Page 4, lines 139-146 and Page 5 lines 162-167)
Objectives	3	State specific objectives, including any prespecified hypotheses (Page 5, lines 167-171)
**Methods**		
Study design	4	Present key elements of study design early in the paper (Pages 13-14, lines 362-364)
Setting	5	Describe the setting, locations, and relevant dates, including periods of recruitment, exposure, follow-up, and data collection (Pages 13-14, lines 362-364)
Participants	6	(*a*) Give the eligibility criteria, and the sources and methods of selection of participants. Describe methods of follow-up (Pages 13-14, lines 364-369)
		(*b*) For matched studies, give matching criteria and number of exposed and unexposed(NA)
Variables	7	Clearly define all outcomes, exposures, predictors, potential confounders, and effect modifiers. Give diagnostic criteria, if applicable (Pages 14-15, lines 373-411)
Data sources/ measurement	8*	For each variable of interest, give sources of data and details of methods of assessment (measurement). Describe comparability of assessment methods if there is more than one group (Pages 14-15, lines 373-411)
Bias	9	Describe any efforts to address potential sources of bias(Pages 16-17, lines 432-442)
Study size	10	Explain how the study size was arrived at (Pages 13-14, lines 362-364)
Quantitative variables	11	Explain how quantitative variables were handled in the analyses. If applicable, describe which groupings were chosen and why (NA)
Statistical methods	12	(*a*) Describe all statistical methods, including those used to control for confounding (Pages 16-17, lines 413-449)
		(*b*) Describe any methods used to examine subgroups and interactions (Page 17, lines 443-446)
		(*c*) Explain how missing data were addressed (Page 17, lines 448-449)
		(*d*) If applicable, explain how loss to follow-up was addressed (Page 16, lines 427-430)
		(*e*) Describe any sensitivity analyses (Pages 16-17, lines 438-442)
**Results**		
Participants	13*	(a) Report numbers of individuals at each stage of study—eg numbers potentially eligible, examined for eligibility, confirmed eligible, included in the study, completing follow-up, and analysed (Page 5, lines 173-177)
		(b) Give reasons for non-participation at each stage (Page 5, lines 173-177)
		(c) Consider use of a flow diagram (NA)
Descriptive data	14*	(a) Give characteristics of study participants (eg demographic, clinical, social) and information on exposures and potential confounders (Page 5, lines 179-182)
		(b) Indicate number of participants with missing data for each variable of interest (Pages 5-7, lines 182-223)
		(c) Summarise follow-up time (eg, average and total amount) (Page 5, lines 173-177)

Outcome data	15*	Report numbers of outcome events or summary measures over time (Pages 6-7, lines 192-215, Figure 1 and Figure 2)
Main results	16	(*a*) Give unadjusted estimates and, if applicable, confounder-adjusted estimates and their precision (eg, 95% confidence interval). Make clear which confounders were adjusted for and why they were included (Pages 7-8, lines 225-233)
		(*b*) Report category boundaries when continuous variables were categorized (NA)
		(*c*) If relevant, consider translating estimates of relative risk into absolute risk for a meaningful time period (NA)
Other analyses	17	Report other analyses done—eg analyses of subgroups and interactions, and sensitivity analyses (Page 8, lines 233-246)
**Discussion**		
Key results	18	Summarise key results with reference to study objectives (Page 8-9, lines 248-258)
Limitations	19	Discuss limitations of the study, taking into account sources of potential bias or imprecision. Discuss both direction and magnitude of any potential bias (Page 12-13, lines 336-350)
Interpretation	20	Give a cautious overall interpretation of results considering objectives, limitations, multiplicity of analyses, results from similar studies, and other relevant evidence (Pages 9-12, lines 259-335)
Generalisability	21	Discuss the generalisability (external validity) of the study results (Pages 12-13, lines 336-343)
**Other information**		
Funding	22	Give the source of funding and the role of the funders for the present study and, if applicable, for the original study on which the present article is based (Pages 1-2, lines 40-48)

*Give information separately for exposed and unexposed groups.

Note: An Explanation and Elaboration article discusses each checklist item and gives methodological background and published examples of transparent reporting. The STROBE checklist is best used in conjunction with this article (freely available on the Web sites of PLoS Medicine at http://www.plosmedicine.org/, Annals of Internal Medicine at http://www.annals.org/, and Epidemiology at http://www.epidem.com/). Information on the STROBE Initiative is available at http://www.strobe-statement.org.
